# A Comprehensive Analysis of Genioplasty in Facial Feminization Surgery: A Systematic Review and Institutional Cohort Study

**DOI:** 10.3390/jcm14010182

**Published:** 2024-12-31

**Authors:** Alexis K. Gursky, Sachin R. Chinta, Hailey P. Wyatt, Maxwell N. Belisario, Alay R. Shah, Rami S. Kantar, Eduardo D. Rodriguez

**Affiliations:** Hansjörg Wyss Department of Plastic Surgery, New York University Langone Health, New York, NY 10016, USA; alexis.gursky@nyulangone.org (A.K.G.);

**Keywords:** facial feminization surgery, genioplasty, sliding genioplasty, reduction genioplasty

## Abstract

**Background:** Facial feminization surgery (FFS) is critical to gender-affirming surgery, consisting of craniomaxillofacial procedures to align facial features with a patient’s gender identity. Central to FFS is genioplasty, which reshapes or repositions the chin; however, limited research exists on genioplasty in FFS. This review and cohort analysis aim to evaluate current practices and outcomes for individuals undergoing FFS with genioplasty. **Methods:** A systematic review included transfeminine individuals undergoing FFS with genioplasty. A retrospective study reviewed FFS cases with genioplasty between 2017 and 2024. Data collected included demographics, imaging, virtual surgical planning (VSP), complications, and patient-reported outcomes (PROs). **Results:** The review included 12 studies with 1417 patients, with 34.2% undergoing genioplasty. The mean age was 37.3 years, and 60.1% were White. Preoperative imaging and VSP were used in 66.7% of studies, 3D-printed cutting guides in 37.5%, and 3D reconstruction in 75.0%. Reduction genioplasty was the most common technique. All PROs indicated high satisfaction, with a 2.67% revision rate. Complications were low (0.55%), with infections being most frequent (0.48%). In the institutional cohort, 351 patients underwent FFS, with 64.4% undergoing genioplasty, significantly higher than in the review (*p* < 0.001). Sliding genioplasty was preferred without preoperative imaging or VSP. Postoperative dissatisfaction was 3.54%, with 0.88% requiring revision (*p* = 0.063). Complication rates (1.77%) were similar to those of the review (*p* = 0.065). **Conclusions:** Genioplasty is important in FFS, with low complication and revision rates and high patient satisfaction. However, gaps remain in validated PROs and technique-specific outcomes. While preoperative imaging and VSP show benefits, comparable outcomes are achievable without them.

## 1. Introduction

In recent decades, gender-affirming surgery (GAS) has become increasingly popular, with procedures increasing by over 500% between 2016 and 2021 [1]. Currently, an estimated 1.6 million individuals in the United States identify as transgender [2]. Facial feminization surgery (FFS) is a crucial aspect of GAS, leveraging craniomaxillofacial procedures to align facial features with the gender identity of transgender and gender-diverse patients [3,4].

A key component of FFS is genioplasty, which includes the surgical repositioning or reshaping of the chin [5]. Typically, biological male chins are taller and broader, with a more pronounced projection and angle. In contrast, feminine chins are softer and less prominent, sometimes with a “V-shaped” contour [4,5,6]. Therefore, the surgical alteration of the chin can have a profound impact on not only facial aesthetics but also gender perception for transfeminine individuals [4]. Various techniques for genioplasty in FFS include sliding osseous for advancement or setback, reduction or reshaping with the removal of bone, or augmentation with bone grafts or implants [7]. Each technique is chosen based on its ability to modify the lower midface and align with the patient’s aesthetic goals, ultimately enhancing facial symmetry and femininity [7].

Despite the extensive literature on FFS, limited studies focus on genioplasty. Previous research has explored isolated genioplasty techniques and outcomes in cisgender patients, characterized by small sample sizes and heterogeneous outcome measures. This systematic review, paired with an institutional cohort analysis, seeks to analyze the role of genioplasty within FFS. By evaluating surgical techniques, preoperative planning tools, and clinical outcomes, we aim to identify effective genioplasty strategies to enhance patient satisfaction and minimize complication rates.

## 2. Methods

### 2.1. Search Strategy and Information Sources

This systematic review followed the Preferred Reporting Items for Systematic Reviews and Meta-Analyses (PRISMA) 2020 guidelines [8]. We searched databases, including Embase, Web of Science, Medical Literature Analysis and Retrieval System Online (MEDLINE), and the search engine PubMed, with no restriction on publication date. The databases were accessed and queried in July 2024. A search was performed using Medical Subject Heading (MeSH) terms and keywords. The search algorithm is reported in Supplemental Table S1. The review protocol was not registered. The resulting studies were uploaded into Covidence, a web-based collaboration software platform that streamlines the production of systematic reviews [9]. The inclusion and exclusion criteria are specified in Table 1.

Eligible studies included transgender women or gender-diverse individuals undergoing FFS with genioplasty. Studies focused on cisgender women or lacking genioplasty outcomes were excluded. Two independent reviewers (A.K.G. and M.B.) performed title, abstract, and full-text screening, with discrepancies resolved by a third reviewer (S.R.C.). The data extracted included patient demographics, medical and surgical history, preoperative planning modality, genioplasty technique, concurrent FFS procedures, and both patient-reported and clinical outcomes. Data were marked as not reported if they were not found in the article. Data of transgender women undergoing FFS without genioplasty were not included in the extraction.

Bias and quality assessments of all included studies were conducted using the Cochrane Risk of Bias in Non-randomized Studies-of Interventions (ROBINS-I) tool, evaluating seven domains: confounding, participant selection, interventions, deviations from intended interventions, missing data, outcome measurement, and reported results [10]. Two independent reviewers (A.K.G. and M.B.) evaluated each study for risk of bias, with a third reviewer (S.R.C.) resolving conflicts. Studies with low or moderate risk of bias were included in the analysis. A study received an overall low-risk rating if all domains scored a low risk. A moderate-risk rating was also assigned if at least one domain was rated of moderate risk, provided there were no critical or severe risk ratings. Studies with critical or severe risk of bias were not included.

### 2.2. Cohort Review and Institutional Surgical Technique

A retrospective review was conducted at our institution, analyzing transfeminine patients who underwent FFS with the senior author between July 2017 and June 2024. This study period encompassed both the recruitment of participants and data collection. Data were collected from institutional medical records, including operative notes, preoperative assessments, and follow-up evaluations.

Patients were included if they were transfeminine women or gender-diverse individuals who underwent FFS during this period. Patients were excluded if they were transmasculine men, cisgender men, or cisgender women; if they underwent non-FFS procedures; or if they had incomplete or missing records. The selection process involved reviewing institutional databases to identify eligible cases. All patients provided informed consent prior to surgery and institutional review board approval was obtained for this study.

The variables of interest included patient demographics, comorbidities, surgical history, self-reported postoperative dissatisfaction, follow-up time, and genioplasty-related complications. To mitigate bias, consistent definitions for variables and outcomes were used to minimize variability in data interpretation. Clearly defined inclusion and exclusion criteria were applied to ensure study homogeneity. Additionally, patients with incomplete or missing records were excluded to avoid potential errors.

The senior author performed sliding advancement genioplasty using an intraoral approach through the gingivobuccal sulcus, extending to the mandibular symphysis. If the patient underwent a concomitant chondrolaryngoplasty, the genial segment was assessed through an extraoral submental incision. The chin prominence was exposed by elevating the gingival periosteum and contoured using a pineapple burr. A full-thickness osteotomy was performed inferior to the bilateral mental nerves, and the bony segment of the chin was advanced and secured with a monocortical plate and screws. The chin’s profile was assessed externally, and additional burring was performed if necessary.

The cohort adhered to the Strengthening the Reporting of Observation Studies in Epidemiology (STROBE) guidelines, addressing all key aspects such as study design, setting, participant eligibility criteria, variable definitions, data sources, statistical methods, and efforts to mitigate bias. This ensured transparency and high-quality data reporting and presentation [11].

### 2.3. Statistical Analysis

All data processing and analyses were performed with R (RStudio version 4.2.3; R Core Team 2023) [12]. All results derived from the systematic review studies were appropriately weighted using the inverse variance method to ensure reliable and accurate statistical comparisons with the institutional cohort. For continuous variables in the systematic review, standard deviations were estimated as the range divided by four, if not reported in the original study. Continuous variables were compared using two-sided independent t-tests, and categorical variables were analyzed using Pearson’s chi-squared test of independence. A *p*-value lower than 0.05 was considered statistically significant.

## 3. Results

### 3.1. Systematic Review

**Figure 1:**
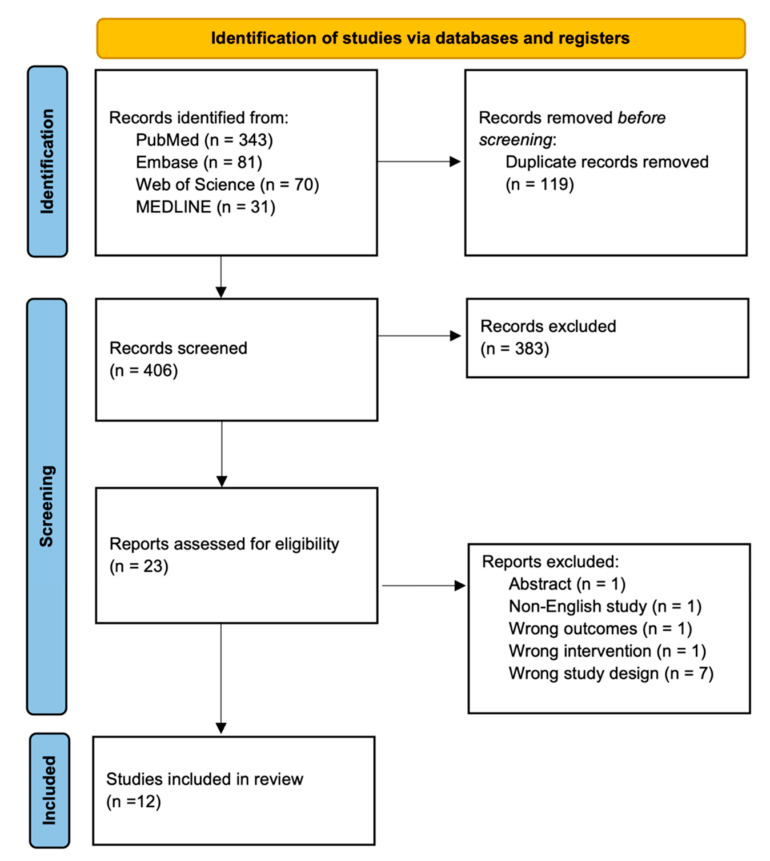
PRISMA flow diagram. The search strategy and article selection based on inclusion and exclusion criteria according to Preferred Reporting Items for Systematic Reviews and Meta-Analyses (PRISMA) 2020 guidelines—12 studies were included in the review.

In adherence to PRISMA 2020 guidelines, the initial search yielded 525 citations. After removing 191 duplicate records, 406 remained for title and abstract screening. After screening, 383 articles were excluded, leaving 23 manuscripts for full-text review. Of these, 11 reports were excluded for being non-English, having the wrong outcomes, or having the wrong study design, ultimately revealing 12 studies available for review (Figure 1).

Using the ROBINS-I tool for risk of bias, eight studies (66.7%) were rated as having a moderate risk. This was primarily due to bias in measuring outcomes since they did not use validated tools to record patient-reported outcomes. The remaining four studies (33.3%) were deemed low risk of bias. No articles were rated as having a serious or critical risk of bias (Supplemental Figure S1).

### 3.2. Study and Cohort Characteristics

As summarized in Table 2, the studies comprised both retrospective and prospective designs, with study periods ranging from 1 to 10 years.

A total of 1417 transgender women underwent FFS, of whom a weighted total of 484 (34.2%) patients underwent genioplasty. All patients in the included studies underwent at least one other facial feminization procedure in addition to genioplasty, such as rhinoplasty, mandibuloplasty, forehead reconstruction, blepharoplasty, and chondrolaryngoplasty. The follow-up ranged from 6 to 110 months.

At our institution, 351 patients underwent FFS, resulting in 226 genioplasties (64.4%), a significant difference from the systematic review (*p* < 0.001). All patients underwent concomitant facial feminization procedures with genioplasty, including malar fat transfer, malar implants, lip augmentation, blepharoplasty, forehead reconstruction, brow lift, rhinoplasty, mandibuloplasty, otoplasty, and chondrolaryngoplasty. The follow-up time ranged between 1 and 42 months.

### 3.3. Patient Demographics and Medical History

The average age in the systematic review studies was 37.3 ± 2.83 years, ranging from 18 to 73 years [15,16,18,19,20,21,22,23]. Most patients were White (60.1%) [16,19,23] and had a history of preoperative hormone use (88.5%) [13,14,15,19,20,21]. Notably, some had a history of GAS of the body (40.2%) [15,16,20,21,22,23] and prior FFS (39.7%) [15,16,20,21], with a smaller fraction having previous genioplasties (4.13%) [15,20,21]. The mean body mass index (BMI) was 26.4 ± 0.28 kg/m^2^ [16,20]. Common comorbidities included psychiatric disorders, current smoking, human immunodeficiency virus/acquired immunodeficiency syndrome (HIV/AIDS), and pulmonary diseases [13,15,16,19,20,21].

The mean age of patients undergoing genioplasty at our institution was 33.8 ± 9.58 years. A significant racial and ethnic distribution difference was observed compared to the reviewed studies (*p* < 0.001), with Black/African-American patients being the most represented (29.2%), followed by Hispanic/Latino and White patients (both 26.5%). The BMI in the cohort paralleled that in the reviewed studies, with an average of 26.4 ± 7.2 kg/m^2^. Hormone use before surgery was almost universal and higher in the cohort at 98.7% (*p* < 0.001). More patients also had GAS of the body before undergoing FFS (52.7%, *p* < 0.001), but a lower proportion of patients had previous FFS (20.8%, *p* < 0.001) performed compared to the review. The number of patients who already had a prior genioplasty was comparable (5.31% vs. 4.13%, *p* = 0.431). Compared to the reviewed studies, a higher proportion of patients were current smokers (27.0% vs. 12.2%, *p* < 0.001), HIV-positive (30.5% vs. 8.38%, *p* < 0.001), had psychiatric disorders (40.3% vs. 10.7%, *p* < 0.001), or pulmonary disease (9.29% vs. 4.16%, *p* < 0.001). Conversely, the systematic review studies reported a higher proportion of alcohol use (58.5% vs. 3.98%, *p* < 0.001) compared to the cohort. Additional patient demographics from the systematic review and cohort study can be found in Table 3.

### 3.4. Preoperative Imaging and Virtual Surgical Planning

**Figure 2:**
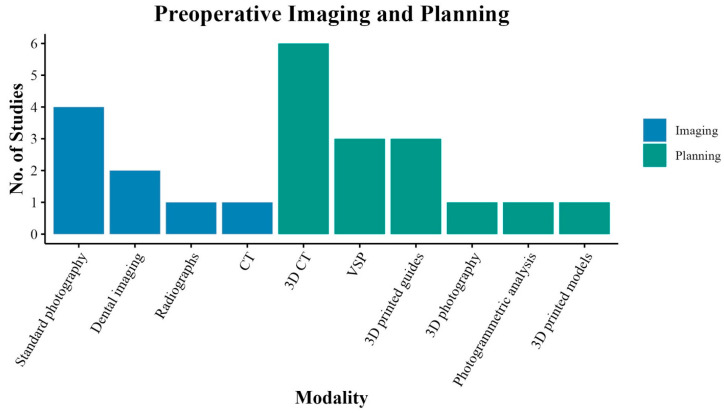
Preoperative imaging and planning for systematic review studies. The number of studies using various preoperative imaging (blue) and planning (green) modalities in facial feminization surgery. *CT, computed tomography; 3D, three-dimensional; VSP, virtual surgical planning*.

Eight studies (66.7%) employed preoperative imaging or virtual surgical planning (VSP). Four studies (50.0%) used standard photography [13,14,15,21]. Computed tomography (CT) with three-dimensional (3D) reconstruction was utilized in six studies (75.0%), aimed at enhanced visualization and surgical planning (Figure 2) [13,14,17,18,20,21]. Custom 3D-printed surgical guides, noted in three studies (37.5%), translated virtual plans into tangible cutting guides [18,19,21]. Uniquely, La Padula et al. utilized 3D printing to create a skull model to help patients better understand the surgery [14].

### 3.5. Genioplasty Surgical Technique and Approach

Nine studies (75.0%) reported their approach or surgical technique for genioplasty in FFS, consisting of reduction, sliding, or augmentation procedures (Table 4).

**Figure 3:**
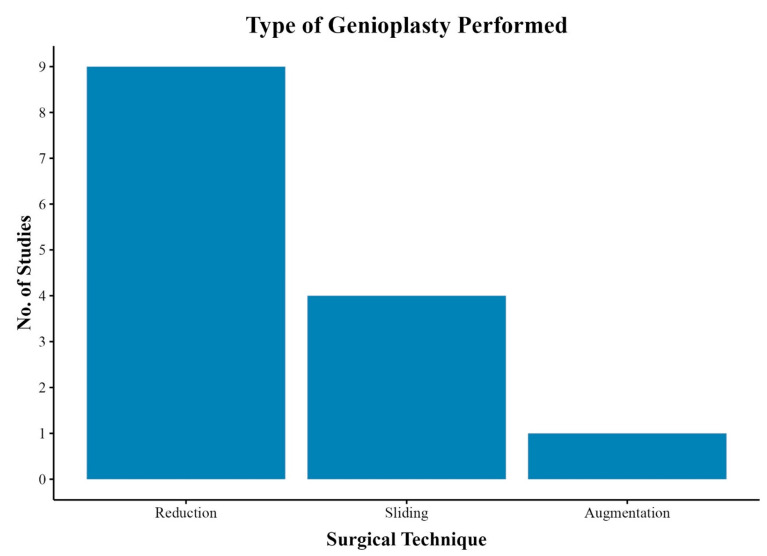
Types of genioplasty performed across studies: reduction, sliding, or augmentation.

Reduction genioplasty was the most commonly employed technique, reported in nine studies [13,14,15,17,18,19,20,21,24]. Sliding genioplasty was detailed in four studies [13,15,20,21]. Lastly, augmentation genioplasty was the least common, with only Simon et al. using interposing mandibular bone grafts for vertical enhancement (Figure 3) [21]. Several studies also incorporated bony burring for additional contouring to achieve a more feminine chin [15,20,21]. The intraoral approach was the most reported [13,18,19,20,21], although Rochlin et al. reported an extraoral approach during concurrent tracheal shave procedures [20].

### 3.6. Overview of Outcomes

**Figure 4:**
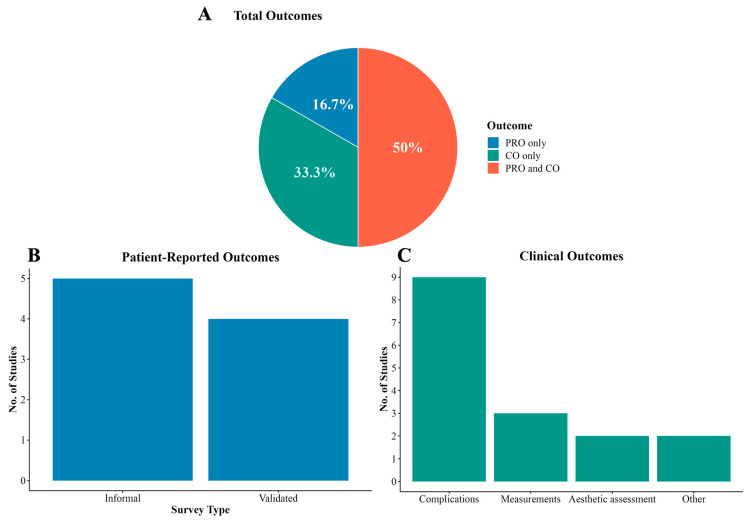
Patient-reported and clinical outcomes from systematic review studies. (**A**) Overview of outcomes reported in systematic studies, PRO only, CO only, or both. (**B**) Number of studies that report PROs, including informal surveys or validated instruments. (**C**) Various clinical outcomes measured, including complications, measurements, or aesthetic assessment. The other categories include surgical planning accuracy and genioplasty revision rate. *PRO, patient-reported outcome; CO, clinical outcome*.

Primary endpoints were categorized into patient-reported (PROs) and clinical outcomes (COs). Six studies (50.0%) reported both (Figure 4A) [13,14,15,17,18,21]. Eight studies reported PROs using informal surveys [13,15,17,18,21] or validated instruments [14,22,23] (Figure 4B). Ten studies reported COs, detailing complications [13,14,15,16,17,18,19,21,24], skull measurements [13,15,17], or aesthetic assessments [13,15] (Figure 4C).

### 3.7. Patient-Reported Outcomes

Eight studies reported PROs: five were informal surveys and four used validated tools. All studies using informal surveys reported improved satisfaction, quality of life (QOL), and femininity perception [13,17,21]. Two studies used the validated instrument, FACE-Q, to evaluate satisfaction regarding specific facial features. Almeida et al. and Alper et al. noted a significant increase in overall satisfaction with facial appearance, 69.3 and 66.8, respectively, and with the chin, 73.0 and 70.2, respectively [22,23]. Alper et al. also used the World Health Organization Quality of Life Scale-Short Form (WHOQOL-BREF) and reported significant improvements in the physical and psychological domains [23]. Other validated tools, including the 36-Item Short Form Health Survey Version 2 Quality of Life (SF-36v2), Satisfaction with Life Scale (SWLS), and Subjective Happiness Scale (SHS), also reported significant postoperative improvements in these domains [14,15]. Details of the PROs and the corresponding studies are provided in Table 5.

Of the 221 genioplasty patients in our cohort, 38 (17.2%) reported dissatisfaction with their outcomes, and 8 patients (3.54%) reported dissatisfaction related to the aesthetic outcomes of their chins.

### 3.8. Clinical Outcomes

The overall complication rate of FFS genioplasty was 0.55% across 1185 patients in the reviewed studies. Major complications included various infections (0.48%) [16,18,19,21] and hematomas (0.61%) [17,21], while the remaining minor complications were sagging skin, witch’s chin, and hypertrophic scarring (Table 6) [15,21].

Infection management typically involved antibiotics, drainage, or return to the operating room for washout or hardware replacement. No permanent nerve damage to the mental or alveolar nerves was reported [17,18,21]. However, temporary paresthesia of the lower lip and chin was reported by Simon et al., but resolved spontaneously within 4 to 30 weeks [21]. A total of 69 patients across three studies reported no complications after FFS [13,14,24]. The revision rate for genioplasty was 2.67%, primarily driven by the need for further feminization and corrections in contouring or reduction [15,20,21].

The retrospective cohort analysis showed similar genioplasty complication rates, with four patients (1.77%) reporting complications, aligning closely with the review (*p* = 0.065). Three patients (1.33%) had infections at the intraoral gingivobuccal incision and were treated with oral antibiotics. The remaining patient experienced a hematoma (0.44%). The distribution of infections and hematoma did not significantly differ from the review findings. Two patients (0.88%) underwent genioplasty revisions, consistent with the review (*p* = 0.063).

Postoperative measurements were another CO captured by four of the reviewed studies. Photogrammetric analysis by Morrison et al. showed significant increases in nasolabial and Frankfort horizontal to mandibular plane angles, consistent with more feminized features [15]. Li et al. demonstrated significant changes in skeletal and soft tissue ratios, with increased zygomatic prominence and reduced gonial width and height [17]. Raffaini et al. reported a mean distance reduction of 0.55 cm between the lower incisors’ superior edge and the mandible’s inferior border [13]. Lastly, Tawa et al. determined the accuracy of preoperative VSP using postoperative CT scans for 26 genioplasty patients, reporting a mean accuracy of 96.2% [18].

Aesthetic assessments were reported in two of the reviewed studies. Raffaini et al. further confirmed the success of FFS, with 87.8% of patients being rated as “very much improved” by independent surgeons who referenced pre- and postoperative photographs and CT scans at 24 months [13]. However, Morrison et al. noted that, while gender appearance and aesthetics scores for their FFS cohort improved, they were still below those of cisgender female controls [15].

## 4. Discussion

Genioplasty is a key component of FFS to achieve facial harmony and enhance femininity [5,7]. Foundational studies have long recognized the transformative effect of chin reshaping in feminizing the face, and our findings validate its importance while reporting current practices and outcomes. Genioplasty was performed in 34.2% of the transgender patients in the reviewed FFS studies and a significantly higher proportion (64.4%) at our institution. The sharp rise in national FFS genioplasty rates from 3.7% to 41.5% between 2016 and 2020 further emphasizes the increasing demand for this procedure and the chin’s critical role in facial feminization [25].

### 4.1. Demographic Insights

Historically, White patients have been the primary demographic undergoing GAS, with Hispanic/Latino and Black/African-American patients being underrepresented compared to the general population [25]. The results of our systematic review support this trend; however, demographic data were provided by only three studies, revealing a gap in understanding racial and ethnic representation in FFS. This limitation raises essential questions about FFS accessibility for marginalized communities who often face compounded socioeconomic barriers. Notably, our institution’s diverse and urban setting showed a different demographic pattern, with Black/African-American patients as the majority in FFS cases. This contrast emphasizes the need for more consistent demographic reporting and the exploration of social determinants of health to address potential inequities in patient care.

### 4.2. Advantages and Challenges of Preoperative Imaging and Virtual Surgical Planning

Over two-thirds of the studies reviewed used preoperative imaging and VSP. Preoperative imaging includes radiographs, CT scans, or dental imaging, while VSP serves as a digital aid to help surgeons pre-plan complex procedures using 3D modeling [26]. These craniomaxillofacial tools have been shown to enhance patient satisfaction by achieving mutual aesthetic goals and refining preoperative measurements without increasing complications [27,28,29]. Custom 3D printing guides have also been shown to improve procedural efficiency and safety. These guides serve as templates for precise bone cutting, helping to prevent injury to nearby structures, such as the mental nerve, during genioplasty [29,30,31]. While VSP can be valuable, its impact may vary by procedure type. One study found that VSP in FFS significantly improved accuracy in forehead reconstruction. However, for the chin, VSP provided minor efficiency, safety, and accuracy improvements compared to cases without preoperative planning [30].

Using VSP presents notable challenges, including cost, time demands, and a steep learning curve [26,32]. While the 3D printing of patient-specific surgical guides can be manufactured at reduced rates, pre- and post-surgical analysis with VSP can dramatically increase the costs incurred by patients and providers [14,26]. For example, VSP in the mandibular reconstruction setting can lead to an additional fee of USD 7099 per patient. Furthermore, leveraging these technologies may or may not lead to enhanced postsurgical outcomes and complication rates [26].

Our institution has developed a streamlined approach using traditional intraoperative planning that meets patient expectations, provides high-quality aesthetic outcomes, and maintains low and comparable complication rates. Thus, integrating VSP as a standard could inadvertently restrict FFS accessibility, particularly for lower-income patients. A nuanced cost–-benefit analysis is needed to determine if VSP justifies broad adoption in FFS.

### 4.3. Genioplasty Techniques and Approaches

Reduction genioplasty, also known as a 3-piece or T-shaped genioplasty, was the most reported technique in our review. This method involves a horizontal osteotomy with one or two vertical cuts, the removal of the central bone fragment, and the joining of both ends of the chin to reduce or increase its projection and narrow it into the desired feminine “V” shape [7,33]. The second most used technique was the sliding genioplasty, which involves the horizontal repositioning of the chin to adjust projection or vertical height. This technique was frequently reported in conjunction with reduction genioplasty, with alterations to the surgical protocol based on patient and physician preference. All sliding genioplasty procedures in the reviewed articles included additional contouring with bony burring.

Our institutional preference is sliding genioplasty with burring to refine the chin. Advancing the chin accentuates the labiomental fold, and burring reduces paramedial prominences while softening the chin’s contour [34,35]. This combination creates a cohesive profile and avoids a step-off deformity seen in osseous reduction [7,35]. Emphasis is placed on optimizing the overall side profile, addressing key areas of the face, including the forehead, nose, chin, and thyroid cartilage. Although this sliding technique was reported less in the systematic review, our cohort has demonstrated comparably low complication, revision, and satisfaction rates.

Despite its technical complexity and increased risk of infection, the intraoral approach was overwhelmingly favored within our cohort for its aesthetic results and avoidance of external scarring. While some surgeons prefer the extraoral approach for better exposure, our review shows it is less commonly reported [36]. Only one study noted using an extraoral approach when performing concurrent tracheal shave procedures, simplifying the surgery and minimizing the number of incisions [20].

Unfortunately, no study differentiated outcomes by specific genioplasty techniques, making it difficult to determine which method yields the best PROs and COs. Future research is needed in this area. However, the studies that only used the reduction technique consistently reported excellent outcomes with few complications and high patient satisfaction [14,17,18,19,24].

### 4.4. Patient Satisfaction and Quality of Life Measures

Validated instruments such as FACE-Q and SF-36v2 QOL provide a more granular understanding of patient satisfaction, which can help surgeons target specific areas for improvement. However, most reviewed studies relied on informal surveys, presenting positive results but lacking details on specific facial features. In the field of GAS, there are limited validated tools specifically for transgender patients, as most were developed for cisgender populations. A meta-analysis found that, although 59.1% of non-genioplasty GAS studies addressed PROs, only 4.3% used tools that were partially validated in transgender patients [37]. The new gender-affirming care survey, GENDER-Q, has been vigorously tested and assesses many facets, including patients’ appearance, quality of life, healthcare experiences, and use of GAS-related devices [38]. Incorporating this questionnaire into future studies will be pivotal for the GAS and FFS community, leading to more impactful and nuanced results. A validated measure focused specifically on FFS would also be highly beneficial. Our retrospective cohort analysis collected dissatisfaction and revision rates, but we aim to incorporate additional PROs to better understand what matters to patients and improve the quality of care. 

### 4.5. Genioplasty Complications and Quantitative Outcomes

Genioplasty-related complication rates in FFS were consistently low across the review. Only 0.55% of patients experienced a complication, primarily infection-related. Genioplasty complications are low, possibly because of minimal soft tissue disruption, simpler access through small, less exposed incisions commonly made intraorally, and predictable anatomy [39]. Our cohort also demonstrated comparably low genioplasty-related complications, with three patients having an intraoral infection and only one with a hematoma.

Infection, although low in incidence, was the most common complication observed across both groups. Hazkour et al. documented a rise in mandibular infections. They implemented preventative measures for all subsequent patients, including increased intraoperative and postoperative antibiotic use, intraoperative betadine application, interlocking sutures for gingival closure, and postoperative chlorhexidine mouth rinses. These adjustments led to a marked decrease in infection rates [19]. Similarly, establishing a standardized institutional protocol for postoperative antibiotic use and wound care in FFS could minimize infection risks and reduce the need for additional returns to the operating room for washouts, improving overall patient safety and consistency in outcomes.

### 4.6. Limitations

This systematic review has several limitations that warrant consideration. First, there is a scarcity of studies reporting detailed demographic data, particularly in racial and ethnic distribution, which limits the ability to assess outcomes across diverse populations. Additionally, the absence of standardized complication reporting for specific facial features complicates comparisons between studies. For instance, some studies grouped complications for genioplasty and mandibuloplasty together or did not link complications to a particular facial procedure. As a result, we are cautious not to overestimate the complications related to genioplasty, which may be lower than reported.

Another limitation was the inability to perform a full meta-analysis, which was precluded by the heterogeneity in the data. To address this, the inverse variance weighting method was used for statistical comparison between groups. Differentiating COs and PROs between various genioplasty techniques was also challenging, as the studies did not provide distinction. This highlights the need for future work to provide more granular insights into which techniques are more favorable and have superior outcomes.

Furthermore, our institutional data, while robust, are based on a single surgeon’s experience in an urban setting, which may limit the generalizability to other populations and geographic regions. Lastly, this systematic review protocol was not registered, which we acknowledge may introduce potential bias. However, we adhered to a strict predefined methodology throughout the systematic review to minimize risk.

## 5. Conclusions

Genioplasty is an essential component of FFS, associated with low complication and revision rates and high levels of patient satisfaction, femininity perception, and quality of life. Despite the high case volume, gaps remain in reporting demographics, validated PROs, and outcomes linked to specific surgical techniques. Preoperative imaging and VSP are widely utilized and have clear benefits. Still, findings from the institutional cohort indicate that equivalent outcomes can be achieved without these tools, raising the question of resource utilization and cost-effectiveness. While our cohort’s sliding genioplasty technique was less frequently utilized in the reviewed studies, it demonstrated comparable complication rates and PROs, suggesting that successful outcomes may depend more on surgical experience than on increasingly complex techniques. Thus, clinical acumen should guide technique choice as the field evolves, focusing on efficacy and patient-centered care.

## Figures and Tables

**Table 1:** Inclusion and exclusion criteria for the systematic review.

	Inclusion Criteria	Exclusion Criteria
Article type	Randomized controlled trials, non-randomized clinical trials, case–control studies, case series (*n* ≥ 5), cohort studies, cross-sectional studies	Systematic reviews, meta-analyses, case series (*n* < 5), case reports, commentaries, conference abstracts, editorials, reviews, unpublished data
Population	Transgender women or gender-diverse individuals undergoing facial feminization surgery	Cisgender women, cisgender men, transgender men, transgender women not undergoing facial feminization surgery, cadaveric studies, animal studies, syndromic patients
Interventions	Genioplasty, surgical correction of the chin, or chin implant procedures aimed at achieving a feminine appearance	Genioplasty, surgical correction of the chin, or chin implant procedures not aimed at achieving a feminine appearance; non-surgical or injectable procedures performed on the chin
Language	English	Non-English

**Table 2:** Included manuscripts and study characteristics.

Author (Year)	Type of Study	Study Period	No. of Patients	Genioplasties *n* (%)	Other FFS Surgeries
Raffaini (2016) [13]	Retrospective	2003–2013	33	33 (100) ^1^	Rhinoplasty, mandibuloplasty, facial prosthesis removal, forehead reconstruction, chondrolaryngoplasty, neck liposuction, face lift, fat grafting, other orthognathic surgery
La Padula (2019) [14]	Retrospective	2015–2018	25	25 (100)	Rhinoplasty, mandibuloplasty, forehead reconstruction, chondrolaryngoplasty, neck liposuction, fat grafting, hairline advancement, lip lift, other orthognathic surgery, malar implant, masseter reduction, angle implants
Morrison (2020) [15]	Prospective	–	66	45 (68.2)	Rhinoplasty, mandibuloplasty, forehead reconstruction, chondrolaryngoplasty, hairline advancement, brow lift, lip lift, blepharoplasty, hair transplant
Chou (2020) [16]	Retrospective	2017–2018	121	102 (84.3) ^1^	Rhinoplasty, mandibuloplasty, forehead reconstruction, chondrolaryngoplasty, neck liposuction, fat grafting, hairline advancement, brow lift, lip lift, blepharoplasty
Li (2021) [17]	Prospective	–	16	16 (100)	Mandibuloplasty and malarplasty
Tawa (2021) [18]	Prospective	2018–2019	45	35 (77.8)	Mandibuloplasty and forehead reconstruction
Hazkour (2022) [19]	Retrospective	2020–2021	31	25 (80.6)	Rhinoplasty, mandibuloplasty, forehead reconstruction, chondrolaryngoplasty, neck liposuction, face lift, fat grafting, hairline advancement, brow lift, lip lift, blepharoplasty, orbit modification
Rochlin (2022) [20]	Retrospective	2017–2021	161	7 (4.34) ^2^	Rhinoplasty, forehead reconstruction, chondrolaryngoplasty, fat grafting, malarplasty
Simon (2022) [21]	Retrospective	2010–2019	837	328 (39.2) ^1^	Rhinoplasty, mandibuloplasty, forehead reconstruction, chondrolaryngoplasty, lip lift, hair transplant, malarplasty
Almeida (2023) [22]	Retrospective	–	23	18 (78.3)	Rhinoplasty, mandibuloplasty, forehead reconstruction, chondrolaryngoplasty, fat grafting
Alper (2023) [23]	Prospective	–	48	39 (81.3)	Rhinoplasty, mandibuloplasty, forehead reconstruction, chondrolaryngoplasty, fat grafting, hairline advancement, brow lift
Pokrowiecki (2024) [24]	Retrospective	2021–2023	11	11 (100)	Mandibuloplasty

*FFS, facial feminization surgery.* ^1^ Includes genioplasties and mandibuloplasties; ^2^ Revision surgeries.

**Table 3:** Patient demographics and past medical history.

	No. of SR Studies	SR Total Sample Size	Weighted SR	Cohort (*n* = 226)	*p*
Age (Years)	7	1211	37.3 ± 2.83	33.8 ± 9.58	0.356
Race or ethnicity					<0.001
White	3	200	120 (60.1)	60 (26.5)	
Multiracial	2	152	20 (13.0)	23 (10.2)	
Black/African American	2	152	8 (5.22)	66 (29.2)	
Hispanic/Latino	1	121	15 (12.4)	60 (26.5)	
Other	1	48	17 (35.4)	0 (0)	
Asian	2	152	11(7.43)	8 (3.54)	
Body mass index (kg/m^2^) ^1^	2	282	26.4 ± 0.28	26.4 ± 7.24	0.993
Hormone use	6	1153	1021 (88.5)	223 (98.7)	<0.001
History of GAS ^2^	6	1256	504 (40.2)	119 (52.7)	<0.001
History of FFS	4	1185	471 (39.7)	47 (20.8)	<0.001
Prior genioplasty	3	1064	44 (4.13)	12 (5.31)	0.431
Comorbidities					
Current smoking	4	1185	144 (12.2)	61 (27.0)	<0.001
Psychiatric or mood disorders	2	282	30 (10.7)	91 (40.3)	<0.001
HIV/AIDS	3	313	26 (8.38)	69 (30.5)	<0.001
Pulmonary disease	2	282	12 (4.16)	21 (9.29)	0.022
Drug use	1	161	63 (39.0)	78 (34.5)	0.352
Cardiovascular disease	1	121	26 (21.5)	9 (3.98)	<0.001
Autoimmune disorder	1	121	6 (4.96)	-	
Diabetes	1	121	5 (4.13)	4 (1.77)	0.187
Alcohol use	1	161	94 (58.5)	9 (3.98)	<0.001
Anticoagulation	1	121	3 (2.48)	-	
None	2	154	59 (38.0)	38 (16.8)	<0.001

*SR, systematic review; GAS, Gender-affirming surgery; FFS, Facial feminization surgery; HIV/AIDS, Human immunodeficiency virus and acquired immunodeficiency syndrome-*. Data reported as mean ± standard deviation or n (%). ^1^ Body mass index was calculated as an unweighted mean. ^2^ Not including FFS procedures.

**Table 4:** Genioplasty approach and surgical technique.

Author (Year)	Approach	Surgical Technique	Type of Genioplasty
Raffaini (2016) [13]	Intraoral	Reduction genioplasty, sliding bone advancement, or simple remodeling	Reduction or sliding
La Padula (2019) [14]		Reduction genioplasty	Reduction
Morrison (2020) [15]		Reduction ostectomy, sliding genioplasty for chin advancement or vertical augmentation, or bony burring	Reduction or sliding
Chou (2020) [16]	-	-	-
Li (2021) [17]		Osteoplastic genioplasty for vertical shortening or narrowing	Reduction
Tawa (2021) [18]	Intraoral	Reduction genioplasty with 3D-printed surgical guides	Reduction
Hazkour (2022) [19]	Intraoral	Osseous reduction genioplasty with 3D-printed surgical guides	Reduction
Rochlin (2022) [20]	Intraoral or extraoral (with concomitant tracheal shave)	Osteotomy to reduce height, advancement genioplasty, bony or silicone contouring with burr, or direct excision of subcutaneous tissue	Reduction or sliding
Simon (2022) [21]	Intraoral	Vertical reduction and sliding genioplasty with 3D-printed surgical guides and ultrasonic microvibration, bone contouring with burr, or vertical increase with interposing mandibular bone grafts	Reduction, sliding, or augmentation
Almeida (2023) [22]	-	-	-
Alper (2023) [23]	-	-	-
Pokrowiecki (2024) [24]	-	Reduction T-genioplasty, including horizontal and M-shape	Reduction

**Table 5:** Patient-reported outcomes in the systematic review studies.

Patient-Reported Outcomes	Studies
Informal surveys	
Satisfaction	Simon et al., Morrison et al., Li et al., Tawa et al. [15,17,18,21]
Femininity perception	Simon et al. [21]
QOL	Raffaini et al. [13]
Validated instruments	
FACE-Q	Almeida et al., Alper et al. [22,23]
WHOQOL-BREF	Alper et al. [23]
SF-36v2 QOL	Morrison et al. [15]
SWLS and SHS	La Padula et al. [14]

FACE-Q, WHOQOL-BREF, SF-36v2 QOL, SWLS, and SHS are all validated instruments. QOL, *quality of life; WHOQOL-BREF, World Health Organization Quality of Life Scale-Short Form; SF-36v2 QOL, 36-Item Short Form Health Survey Version 2 Quality of Life; SWLS, Satisfaction with Life Scale; SHS, Subjective Happiness Scale*.

**Table 6:** Genioplasty-related complications.

Complication	No. of SR Studies	SR Total Sample Size	Weighted SR	Cohort (*n* = 226)	*p*
Any complication	9	1185	7 (0.55)	4 (1.77)	0.065
Infection	4	1034	5 (0.48)	3 (1.33)	0.148
Unspecified infection ^1^	2	882	3 (0.37)	3 (1.33)	
Abscess	1	31	2 (6.45)	0 (0)	
Osteomyelitis	1	31	1 (3.23)	0 (0)	
Phlegmon	1	121	1 (0.83)	0 (0)	
Hematoma ^1^	2	853	5 (0.61)	1 (0.44)	0.796
Sagging skin	1	205	11 (5.37)	0 (0)	<0.001
Witch’s chin or iatrogenic jowling	1	66	2 (3.03)	0 (0)	0.009
Hypertrophic scar	1	66	1 (1.52)	0 (0)	0.064
Nerve damage	3	898	0 (0)	0 (0)	
None	3	69	69 (100)	222 (98.2)	0.266

Data reported as n (%). ^1^ Includes patients who also underwent mandibuloplasty. *SR, systematic review.*

## Data Availability

The original contributions presented in the systematic review are included in the article/supplementary material. Further inquiries can be directed to the corresponding author. Additionally, institutional cohort data presented in this study are available on request from the corresponding author due to privacy and ethical restrictions.

## Supplementary Materials

The following supporting information can be downloaded at: https://www.mdpi.com/article/10.3390/jcm14010182/s1. Table S1: Search algorithm for literature search; Figure S1: Quality and bias assessment using Cochrane ROBINS-I.
