# Supplementary material for: A Comprehensive Analysis of Genioplasty in Facial Feminization Surgery: A Systematic Review and Institutional Cohort Study
Source: J Clin Med. 2024 Dec 31;14(1):182. doi: 10.3390/jcm14010182 (PMC11721636; doi:10.3390/jcm14010182)
Supplement: Supplementary file 1 [file jcm-14-00182-s001.zip › jcm-3362081-supplementary/supplementary Table S1.pdf]

Supplemental Table 1. Search algorithm for literature search

Search algorithm
(("Facial feminization surgery" OR FFS OR "Feminization"[MeSH])<br>AND<br>("Genioplasty"[MeSH] OR "Chin"[MeSH] OR Mentoplasty OR "FFS genioplasty" OR "Feminizing genioplasty"))
