# Supplementary figures and images for: A Comprehensive Analysis of Genioplasty in Facial Feminization Surgery: A Systematic Review and Institutional Cohort Study
Source: J Clin Med. 2024 Dec 31;14(1):182. doi: 10.3390/jcm14010182 (PMC11721636; doi:10.3390/jcm14010182)

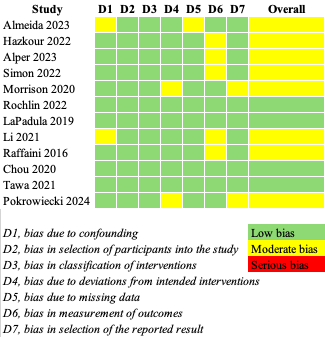

Supplement: Supplementary file 1 [file jcm-14-00182-s001.zip › jcm-3362081-supplementary/Supplemental Figure S1.png]
